# Retracted: Utilization of Maternal and Child Health Care Services by Primigravida Females in Urban and Rural Areas of India

**DOI:** 10.1155/2015/125498

**Published:** 2015-01-19

**Authors:** International Scholarly Research Notices

The paper titled “Utilization of Maternal and Child Health Care Services by Primigravida Females in Urban and Rural Areas of India” [1], published in International Scholarly Research Notices, has been retracted as it is found to contain a substantial amount of material, without referencing, from the dissertation titled “A Comparative Study to Assess the Availability and Utilization of Reproductive and Child Health Services between Rural and Urban Antenatal Care Set Up in Maharashtra with Assessment of Effect of Close Supervision and Support in Selective Subjects in Both Groups,” by Nitin Pandurang Mahajan and Abhiram M. Kasbe.
